# Federated Learning Attacks Revisited: A Critical Discussion of Gaps, Assumptions, and Evaluation Setups

**DOI:** 10.3390/s23010031

**Published:** 2022-12-20

**Authors:** Aidmar Wainakh, Ephraim Zimmer, Sandeep Subedi, Jens Keim, Tim Grube, Shankar Karuppayah, Alejandro Sanchez Guinea, Max Mühlhäuser

**Affiliations:** 1 Telecooperation Lab, Technical University of Darmstadt, 64289 Darmstadt, Germany; 2 National Advanced IPv6 Centre (NAv6), University of Science Malaysia, Penang 11800, Malaysia

**Keywords:** federated learning, inference attacks, poisoning attacks, systematic mapping study

## Abstract

Deep learning pervades heavy data-driven disciplines in research and development. The Internet of Things and sensor systems, which enable smart environments and services, are settings where deep learning can provide invaluable utility. However, the data in these systems are very often directly or indirectly related to people, which raises privacy concerns. Federated learning (FL) mitigates some of these concerns and empowers deep learning in sensor-driven environments by enabling multiple entities to collaboratively train a machine learning model without sharing their data. Nevertheless, a number of works in the literature propose attacks that can manipulate the model and disclose information about the training data in FL. As a result, there has been a growing belief that FL is highly vulnerable to severe attacks. Although these attacks do indeed highlight security and privacy risks in FL, some of them may not be as effective in production deployment because they are feasible only given special—sometimes impractical—assumptions. In this paper, we investigate this issue by conducting a quantitative analysis of the attacks against FL and their evaluation settings in 48 papers. This analysis is the first of its kind to reveal several research gaps with regard to the types and architectures of target models. Additionally, the quantitative analysis allows us to highlight unrealistic assumptions in some attacks related to the hyper-parameters of the model and data distribution. Furthermore, we identify fallacies in the evaluation of attacks which raise questions about the generalizability of the conclusions. As a remedy, we propose a set of recommendations to promote adequate evaluations.

## 1. Introduction

Machine learning (ML) is an approach to imitate the human way of learning. With the help of training data, an ML system is able to learn and recognize patterns in the data with improved accuracy over time. This so-called model can later be applied to other unknown sets of data to make classifications or predictions without requiring human interactions. ML is increasingly used to improve services in many domains and settings, e.g., image processing [1], security [2,3], and smart cities [4,5,6]. Most recently, researchers have developed and improved MCUNet [7], which enables edge devices such as smartphones and sensors to train machine learning (ML) models, and thus makes deep learning accessible in Internet of Things (IoT) settings.

The conventional training approach of ML models is centralized, where large datasets are collected from users and processed by central service providers. These datasets can contain sensitive user information (e.g., health metrics or geographic locations). Therefore, this conventional approach raises users’ concerns about their privacy [8]. Furthermore, the large number and distribution of sensors and IoT devices make it almost impossible to collect and then process the massive and continuous amount of sensor data in a ML model.

Federate learning (FL) is an emerging ML setting that enables multiple entities (clients) to train a joint model while keeping their data locally on their devices. This setting still involves a central server that coordinates the training process by collecting and aggregating model updates from clients to obtain one global model. As the raw data of the clients do not leave their devices, federated learning (FL) is believed to provide privacy benefits [9]. Other interesting security and privacy enhancements for IoT devices also exist, e.g, refs [10,11,12,13,14,15].

However, the distributed nature of the training process in FL has created a new attack surface, where potentially malicious clients can actively participate and adversely affect the training process. The attacks might target either the integrity of the model (e.g., poisoning attacks [16,17]) or the confidentiality of the client training data (e.g., model inversion attacks [18,19]). Recently, researchers have been extensively investigating vulnerabilities in FL and proposing potential attacks. Consequently, the number of publications on attacks against FL remarkably increased; this increase raises serious concerns about the robustness and privacy in FL. Some strong claims even exacerbate these concerns by stating that “federated learning is fundamentally broken” [18], or that some attacks are reaching 100% accuracy [16].

However, a number of these attacks are applicable only under specific conditions and assumptions. For example, some attacks are effective on Neural Network (NN) models only when the training batch size equals 1 (e.g., [20]), or when a special distribution of data among clients is applied (e.g., [18]). In many cases, such assumptions do not hold in real-world deployments. Thus, the applicability of such attacks is questionable. In addition, several attacks are evaluated with limited or impractical setups. For instance, some attacks are evaluated using oversimplified datasets (e.g., [21]) or simplified NN models (e.g., [22]). This in turn affects the generalizability of the experiments, results, and conclusions. A recent work by Shejwalkar et al. [23] fueled this discussion by demonstrating that, contrary to the common belief, FL is highly robust against several attacks in the literature—even without applying any defenses—under practical considerations. Considering the aforementioned issues, the severity and the scope of the vulnerabilities discussed in the literature must be further studied under realistic setups.

In this study, we present a quantitative analysis of attacks against FL via a systematic mapping study (SMS). We first identify research trends that indicate the growth of the field; the properties of the research community, such as affiliations; and targeted publication venues. Then, we provide a structured overview of the attacks with two classification schemes that are based on:

(1) the properties of the attacks, and
(2) the choice of experimental setups used to evaluate the attacks.

We analyze the distribution of publications among the defined attack classes and derive the foci and gaps in the research landscape. Next, we highlight several issues in the assumptions made in some of the works and their implications for the applicability of the attacks. Finally, we identify common fallacies in the evaluation setups and the impacts of these fallacies on the generalizability of the results. To the best of our knowledge, we are the first to conduct such a systematic literature review, which allowed us to thoroughly assess the literature of attacks against FL. Our work shows that each of the studied papers has at least one of the assumption issues or suffers from one fallacy. Notably, several fallacies affect the majority of the papers. Our contributions can be summarized as follows.

- We recognize three research gaps that raise questions about the effectiveness of attacks against specific ML functions (e.g., clustering and ranking) and models (e.g., Recurrent Reural Networks and Autoencoders).
- We highlight three recurring assumptions that limit the applicability of the proposed attacks to real-world deployments. These assumptions are related to the hyper-parameters of the ML model, the fraction of malicious clients, and data distribution.
- We identify six fallacies in the evaluation practices that can cause overestimation of the attacks’ effectiveness. The main fallacies stem from the choice of: datasets, models, and the size of the client population. In addition, we also propose a set of recommendations to mitigate these fallacies.

We would like to emphasize that our work does not negate the existence of vulnerabilities in FL, and thus the security and privacy risks. It also does not undermine the importance of the research on attacks in FL. Rather, it is an attempt to help researchers clarify the scope of these attacks by reflecting on the assumptions and evaluation practices.

The remainder of this paper is organized as follows. In Section 2, we present a background on NN and FL, a summary of related review studies, and an introduction to SMSs. Then, in Section 3, we elaborate on the methodology of our study. In Section 4, we present the results of the mapping process. Subsequently, we further analyze our results and discuss their implications in Section 5. Finally, we conclude the paper in Section 6.

## 2. Background

In this section, we provide the foundations of Neural Networks (NNs) as some of the dominant models in the literature of our field. Then, we introduce FL, along with some core insights about FL’s security and privacy-related issues. Next, we present a summary of multiple review studies that elaborate on these issues. Lastly, we introduce the objectives and methodology of systematic mapping studies (SMSs).

### 2.1. Neural Networks

NNs are a subset of ML algorithms. An NN is comprised of layers of nodes (neurons), including an input layer, one or more hidden layers, and an output layer. The neurons are connected by links associated with weights W. The NN model can be used for a variety of tasks, e.g., regression analysis, classification, and clustering. In the case of classification, for example, the task of the model $\hat{f}$ is to approximate the function f(x)=y, where *y* is the class label of the data sample *x*. To fulfill this task, the model is trained by optimizing the weights W using a loss function *L* and training data consisting of input data $x_{i}$ and corresponding labels $y_{i}$ in order to solve

(1) $$\min_{W}\sum_{i=1}^{N}L_{W}\left(x_{i},y_{i}\right),$$

where *N* is the number of data instances, and $L_{W}$ is the loss corresponding to the weights W. Minimizing the loss function can be achieved by applying one of the optimization algorithms. Gradient descent is one of the basic optimization algorithms for finding a local minimum of a differentiable function. This algorithm is based on gradients ∇W, which are the derivatives of the loss function with regard to the model weights W. The core idea is to update the weights through repeated steps *t* in the opposite direction of the gradient because this is the direction of the steepest descent.

(2) W(t+1)=W(t)−η∇W,

where η is the learning rate, which defines how quickly a model updates its weights. An extension of gradient descent, called minibatch stochastic gradient descent, is widely used for training NNs. This algorithm takes a batch of data samples from a training dataset to compute gradients ∇W and subsequently updates the weights. The batch size *B* is the number of data samples given to the network, after which the weight updating happens. The number of epochs *E* is the number of times the whole training dataset is shown to the network while training. NN models can have different architectures that serve different purposes. Here, we introduce three main architectures.

**Multilayer Perceptron (MLP).** Known also as a feedforward network. This is a general-purpose network that contains fully connected layers of neurons.

**Convolutional Neural Network (CNN).** This architecture is mainly used to detect patterns in the data. It contains convolutional, pooling, and fully connected layers. In the convolutional and pooling layers, a neuron receives input only from a limited number of the neurons in the previous layer.

**Recurrent Neural Network (RNN).** This network operates on sequential data and times series data. It is distinguished by its recurrent structure, which memorizes information across layers. That is achieved by neurons connected in a loop, i.e., using input from prior neurons to influence the current input and output of the neurons.

### 2.2. Federated Learning

Federated learning (FL) is a distributed machine learning setting where *N* entities (clients) train a joint model under the coordination of a central server [24]. The training process starts with the server initializing a model, then goes through several rounds of training (also known as the communication round); each training round *t* consists of the following steps:

1. The server samples a subset of users $K_{t}\ll N$ clients to participate in the training round;
2. The server disseminates the model and training algorithm to the selected clients;
3. Clients train the model locally on their own data;
4. Clients share (only) the resulting gradients (or model updates) with the server;
5. The server aggregates the gradients to derive a new updated global model as follows
  (3) $$W\left(t+1\right)=W\left(t\right)-\eta \sum_{k=1}^{K_{t}}\frac{n_{k}}{n}\nabla W_{k},$$
  where $n_{k}$ is the number of data samples of user *k*, and *n* is total number of data samples.

FL is mainly employed for large-scale applications (cross-device FL), where a massive number of clients participate in training the joint model. As the clients typically have varying amounts of data, the training data are unbalanced. Moreover, the data of a specific client is typically generated based on that client’s activity, which does not necessarily represent the distribution of the data of all clients [9]. In the literature, it was demonstrated that FL is robust to these characteristics and can effectively lead to model convergence [9].

**Figure 1:**
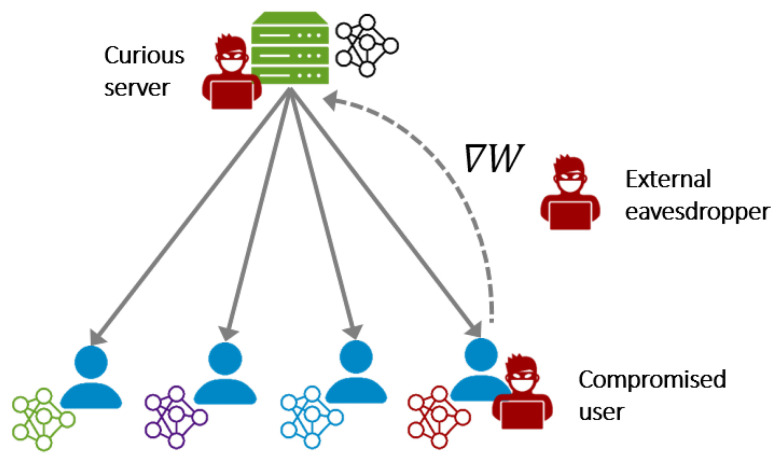
Federated learning overview with three potential adversary access points (in red): curious server, compromised client, and external eavesdropper. The gradients (or models updates) are generated by individual users and shared with a central server that aggregates all the gradients into a global model.

Unlike centralized machine learning, where the clients’ data need to be collected at a central server, FL allows clients to maintain their data locally, while training the model in a distributed manner. By that, FL is claimed to mitigate some privacy risks; however, the distributed nature of FL leads to increasing the attack surface (see Figure 1). For the most part, there are three potential attack points.

**Curious server.** A central server coordinates the training process and performs the core functionality of FL (as shown in the aforementioned 5-step workflow). Such a scheme concentrates all the control in the hands of a sole entity, the server. From a security and privacy perspective, this centralization of control can be seen as a major weakness. This is because using services based on FL forces clients to trust the server to perform the FL functions correctly and apply best privacy practices to protect their updates. In this regard, if the server is malicious, various attacks can be carried out against the clients (e.g., reconstruction attacks [19,25]). More details on the privacy implications of the centralized coordination in FL can be found in [26].

**Compromised client.** Furthermore, the distributed nature of FL and involving the clients actively in the training process open the door for attacks launched by malicious clients. In FL, the model is sent to the clients’ devices, where the clients are typically granted full access to the model’s parameters. This access privilege, in turn, amplifies the malicious clients’ capabilities and enables them to perform sophisticated attacks. These attacks may target the model (e.g., poisoning attacks [17]) or the privacy of the participating clients (e.g., membership inference attack [27]).

**External eavesdropper.** It was believed in the past that the gradients shared between the server and clients do not leak information about the client training data [19]. However, recent attacks demonstrated that external eavesdroppers who have access to these gradients can reconstruct the client data (e.g., leakage from gradients attacks [19,20]).

### 2.3. Review Studies

Several studies in the literature provide overviews of the privacy and security issues in FL, either as a part of general analysis for ML applications or as specific analyses of the FL setting.

**Privacy and Security in ML.** Due to the growing recognition of the threats that ML systems might face, many researchers have presented surveys and systematization of these threats, such as Papernot et al. [28] Al-Rubaie et al. [29], De Cristofaro [30], Rigaki et al. [31], and Zhang et al. [32]. Mainly, these studies focus on the privacy aspect in centralized training and only address FL to a limited extent. Other surveys tackle the privacy and security of deep learning models especially, namely, the works of Mirshghallah et al. [8] and Liu et al. [33].

**Privacy and Security in FL.** In our study, we aim to

(1) structure the publications dealing with privacy and security FL attacks according to classification schemes, thereby providing a structured overview of the research field,
(2) conduct a quantitative analysis of the publications, highlighting areas of focus and gaps in the literature, and
(3) provide a critical discussion of the applicability of the proposed attacks by taking a closer look at their assumptions and evaluation setups.

There are studies in the literature with partially overlapping goals. Enthoven et al. [34] presented a structured overview about attacks and defense mechanisms in FL, but only for privacy attacks against deep learning models. Lyu et al. [35] elaborated additionally on security attacks and pointed out weaknesses in current countermeasures through a qualitative analysis of the literature. However, this study neither provides a quantitative analysis nor discusses the applicability of the attacks. A concise taxonomy of attacks in FL was introduced by Jere et al. [36]. Although the proposed taxonomy is well thought out and justified, the study considers only a small portion of the attacks available in the literature, without discussing their quantity or applicability. Kairouz et al. [24] presented an extensive report of open issues and challenges in FL, including privacy and security issues. However, the extent to which these issues are applicable in real-world scenarios is discussed only briefly. Similar but less comprehensive surveys in terms of the level of detail were also published [37,38].

The aforementioned studies provide very valuable insights into the privacy and security in ML and FL by summarizing and systematizing the existing research in this field. However, none of them have met all of our goals, and all of them have (largely) failed to meet two of our main goals, namely, quantitative analysis and discussion of attack applicability.

### 2.4. Introduction to Systematic Mapping Studies

Systematic mapping [39,40] is a secondary study method that establishes classification schemes and structures in a research field. The analysis of the study focuses on the frequency of publications in each of the defined categories. Such an analysis provides valuable insights into the progress, foci, and gaps of the research field. These insights are not covered by the commonly used systematic literature review (SLR) method, which focuses on surveying primary studies to collect evidence concerning existing solutions [41], while overlooking the frequency of publications. The goals of SMSs include [40]:

(1) providing classification(s) and a taxonomy,
(2) identifying research gaps in the existing literature and possible future research, and
(3) identifying trends and future research directions.

**Figure 2:**
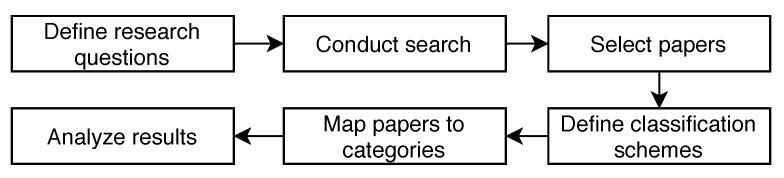
Workflow of the systematic mapping study consists of six steps.

To conduct an SMS, the following steps are taken (see Figure 2):

1. Define a set of research questions to be answered by the analysis of the study.
2. Conduct a search to find the relevant papers.
3. Refine the selection of papers by employing inclusion and exclusion criteria.
4. Define classification schemes to structure the papers into categories.
5. Map the papers to the defined categories.
6. Answer the research questions by analyzing the frequency of papers appearing in the defined categories.

SMSs are widely used in medical research [39] and also gained traction in the field of software engineering (e.g., [42,43]).

## 3. Method

Petersen et al. [39,40] proposed a guideline for conducting SMSs, which serves as a basis for our study outlined in this section.

### 3.1. Objectives and Research Questions

The ultimate goal of our study is to analyze past research and to guide further research on attacks for FL with regard to practical relevance. This concerns, in particular, the effectiveness of attacks, the question of how realistic the assumptions are, and the fallacies observed. As a basis, we analyzed the attacks proposed in 48 scientific papers identified as pertinent for the SMS (see Section 3.2 and Section 3.3). In light of the general goals of the study as set forth in Section 2.3, we based our systematic work on the following three guiding questions.

1. What are the research trends in the domain? For this question, we look at the development of the number of papers over years, the communities that conduct the research, and the type of research.
2. What are the different types of attacks carried out against FL? We identify the attacks that have been proposed in FL and their properties.
3. Which are the evaluation setups commonly used in the literature? We determine common evaluation practices in the field and discuss their implications.

Answering the aforementioned questions requires two sub-steps:

(1) identifying and capturing the research trends, attack types, and evaluation setups in terms of categories and characteristics, and
(2) analyzing the literature and mapping it according to the established categories and characteristics of the respective research trends, attack types, and evaluation setups.

### 3.2. Search Strategy

**Figure 3:**
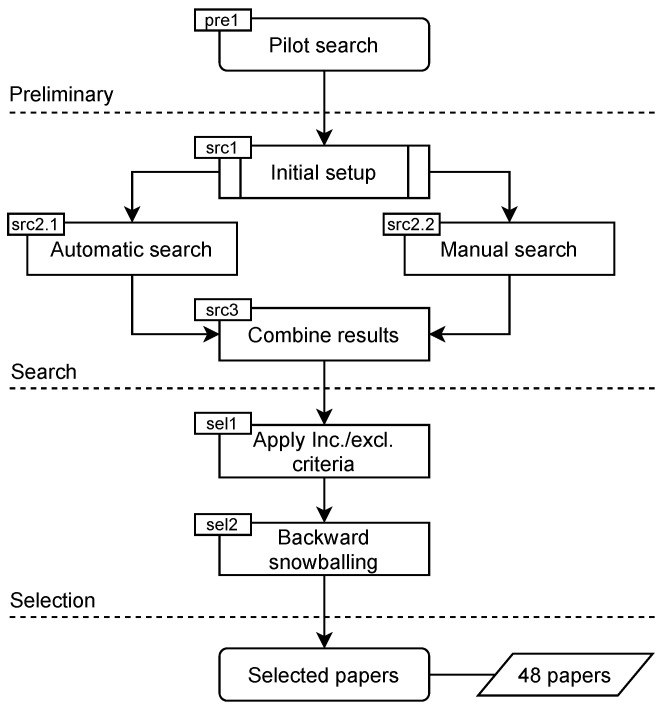
Search and selection process of our SMS. We started with a pilot search as a preliminary step. Then, we conducted the main automatic and manual search. Lastly, we selected the relevant papers.

To set up the main search process, a brief pilot search (pre1 in Figure 3) was carried out, by which an initial set of relevant papers was collected. These papers allowed us to determine relevant keywords and search terms and suitable venues to target in the main search process, and to identify a set of well-known authors in this research area. For this pilot search, Google Scholar was used, since it is one of the indexing databases that covers a large number of publishers. Several search queries were applied using combinations of keywords, which have shown to be important in the research field at hand, including “federated learning”, “privacy attack”, and “security attack”.

The main search phase is presented below, which consisted of two procedures: automatic and manual search.

1. Automatic Search (src2.1 in Figure 3): We conducted the automatic search by relying on several popular search engines, namely, ACM Digital Library, IEEEXplore, Google Scholar, and arXiv. ACM Digital Library and IEEEXplore were considered. as they cover the key research communities (i.e., ML and security communities) and most cited publications from ACM and IEEE computer society. In addition, Google Scholar was used to ensure comprehensive results and avoid any bias towards specific publishers. Furthermore, using arXiv helps to cover the most recent advancements, which are not yet accepted for publication. The keywords to include FL-related terms are: “federated learning” and “collaborative learning”. Additionally, precise keywords related to attacks, namely, “inference attack”, “privacy attack”, and “poisoning attack” were also added to the search. Thereafter, a search string was composed using the updated keywords. This search retrieved all the papers that contain the search string in any part of them, i.e., title, abstract, or body. This in turn might have led to many papers that mention the relevant terms but do not fall within the scope of our study. Such papers werer filtered out in a subsequent step (selection process). The results of the automatic search are shown in Table 1.
2. Manual Search (src2.2 in Figure 3): The titles of the papers published in a set of selected journals and conferences were manually reviewed. The journals and conferences were chosen based on the results of the pilot search to cover all the venues where papers on attacks in FL are published. The complete list of sources is shown in Table 2. As a complementary procedure, a number of well-known researchers in the field (e.g., H. Brendan McMahan) were identified, and their publications (on Google Scholar, private webpages, and university webpages) were tracked. The manual search resulted in identifying 20 potentially relevant articles.

**Table 1:** Automatic search results and search strings.

Database & Papers Found	Search String
Google Scholar: 1.070	(“federated learning” OR “collaborative learning”) AND (“inference attack” OR “privacy attack” OR “poisoning attack”)
ACM Digital Library: 49	
IEEEXplore: 171	
arXiv: 91	

The combined total number of papers gathered from the automatic and manual searches was 756. These papers were found to contain our search strings or to have relevant titles. However, this was not sufficient to consider them in our study. Therefore, it was crucial to specify strict criteria to select the relevant papers among them.

### 3.3. Selection Process

As depicted in Figure 3, after the search, the selection process was conducted. This process consisted of two steps:

(1) applying inclusion and exclusion criteria, and
(2) performing a complementary forward and backward snowballing search.

The following inclusion and exclusion criteria were applied to titles and abstracts. In those cases where the title and abstract did not provide enough information, the body of the paper was considered.

**Inclusion criteria.**

(1) Papers discuss attacks in FL. These papers include those that introduce novel attacks and papers that review existing attacks.
(2) Papers published between 2016 (the year of coining the term Federated Learning by McMahan et al. [9]) and 2021.

**Exclusion criteria.**

(1) Posters.
(2) Papers about FL that do not cover any attack.
(3) Papers not written in English.
(4) Papers not accessible in full-text.
(5) Duplicate papers.

After filtering the papers based on the inclusion and exclusion criteria, forward and backward snowballing techniques were applied. Forward snowballing identifies papers that have cited the papers found in the search phase. As the majority of the papers selected after filtering are recent (2019–2020), they have not yet been cited by many other papers. Therefore, the focus was more on backward snowballing (sel2 in Figure 3). By this technique, the lists of references in the selected papers were reviewed, and the relevant papers were added to our list. Applying this technique resulted in adding only a few more new papers, as our automatic and manual searches already covered almost all the relevant sources.

The final number of papers considered for our analysis was 48. It is worth mentioning that after applying the inclusion and exclusion criteria, there was a remarkable drop in the number of papers. That is because there is a huge number of papers that refer to FL and its attacks but do not contribute to this topic.

### 3.4. Information Extraction and Classification

Each of the final selected papers was examined in detail to extract information on the research trends, attack types, and evaluation setups. This information was utilized to

(1) propose an initial set of classification schemes, and
(2) sort the papers accordingly.

Then, we iterated, after a first orientation path, over these two steps multiple times to refine the classification schemes and remap the papers to the defined categories. In the following section, three main classification schemes are presented that correspond to our guiding questions.

## 4. Results of the Mapping

In this section, we elaborate on the classification schemes and the results of paper mapping. The frequency of papers in each category is presented as the exact number of papers and the percentage with respect to the total number of papers (48). The results are also provided in detail in Table 3 and Table 5. This section is structured along with the defined research questions (see Section 3).

### 4.1. Research Trends

We investigate the trends in the research field through four aspects, namely: the year of publication, the affiliations of the researchers, the venues they targeted to publish their works, and lastly, the type of research conducted according to Wieringa et al. [44].

**Figure 4:**
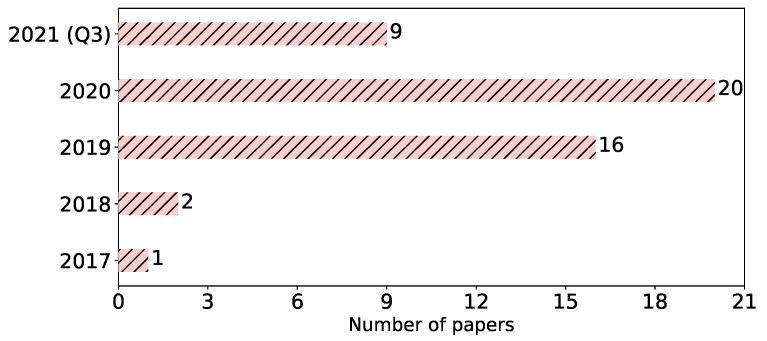
Number of papers per year.

**Year of publication.** In Figure 4, we see the publication years for the studied papers. The first attack against FL was published in 2017 by Hitaj et al. [18]. In the following years, the number of attacks remarkably increased. That reflects the growing attention towards FL in general and its privacy and security issues, in particular. This considerable number of attacks can also be seen, for an external observer, as an indication of the abundance of FL vulnerabilities. Overall, FL is a hot topic, and the number of its applications is growing; therefore, investigating its weaknesses becomes crucial, and this likely will lead to more studies on attacks in the upcoming years.

**Author affiliations.** Our mapping study illustrates that most of the attacks (41 i.e., 85%) come from academia. This could be because researchers in academia freely explore the possibilities to hack technologies and then propose mitigation measures, whereas industry tends to focus on making their services more robust and secure. That is evident from the substantial number of papers on defense mechanisms from industry, especially Google [45,46,47], whereas a smaller number of attacks (2 i.e., 4%) were proposed by industry. Joint projects between academia and industry also exist with 5 (10%) papers.

**Figure 5:**
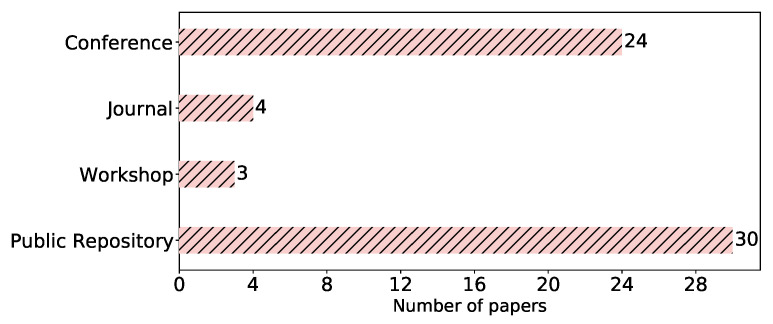
Venue types of the selected papers.

**Venue type.** In our study, we took into account peer-reviewed venues (journals, conferences, and workshops) as shown in Table 2, in addition to public repositories (arXiv). The papers’ distribution among these venues is depicted in Figure 5. We can see the tendency of the community to push their studies to public repositories, where 30 (62%) of the papers were found. This can be due to the fast pace of publications in this field, which urges researchers to share their ideas and results promptly as preprints. Out of these, 13 (25%) were simultaneously published in a peer-reviewed venue, mainly conferences. After arXiv, the conference papers came first, totaling 24 (50%). The low number of publications in journals might be a result of the novelty of the FL concept and the rapid development of its attacks.

**Research type.** To identify the research characteristics in this field, we categorized the papers based on the type of conducted research. We adopt the research types proposed by Wieringa et al. [44].

- Solution: Proposes an approach to solve a problem. The approach can be novel or improves on existing ones. The proposed approach should be supported by good arguments or by other means.
- Validation: Investigates the validity of a novel approach that has not yet been “realized”. The validation can be performed through experiments, simulations, mathematical proofs, etc.
- Evaluation: Studies the properties of an existing approach (analyze, assess, and evaluate) to achieve a better understanding of its potential and limitations.
- Philosophical: Provides new insights, a new way of thinking, or a new conceptual view of research.
- Opinion: States the authors’ position towards a specific topic without introducing any research results.
- Experience: Describes the personal experience of the authors in conducting “a practice”.

Our mapping shows that the studied papers fall into only two categories, namely, Solution and Evaluation, with 37 (77%) and 11 (23%), respectively. On the one hand, the novelty of this research field could be a reason for the abundance of papers within the Solution category, since many privacy and security aspects need to be addressed. On the other hand, this novelty may explain the absence of papers from other categories, such as Experience—it typically requires more time to put the research approaches into practice and develop experience in the domain.

### 4.2. Attack Types

**Figure 6:**
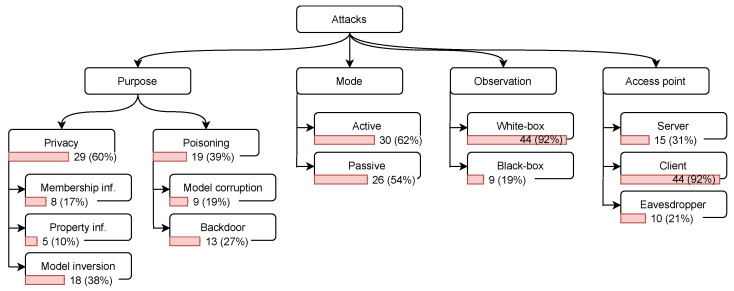
Attack classification with the papers’ distribution. The percentage is with regard to the total number of papers, 48. Most categories are not exclusive; therefore, the papers might sum up to more than 48. More details on the individual papers can be found in Table 3.

To identify the types and properties of these attacks, we consider several aspects, namely, the attack’s purpose, mode, observation, and access point [27,30]. Next, we introduce the common attack categories with respect to each of the aspects. The distribution of publications among these categories is depicted in Figure 6.

**Purpose.** The attack’s purposes can be classified into two main categories.

1. Privacy attacks (inference attacks): These attacks extract information about the training dataset, i.e., user data [30], and fall into three groups based on the obtained information:
  - Membership inference: The adversary aims to determine whether a particular individual (or a data record) belongs to the training dataset [48].
  - Property inference: The adversary aims to infer features of the training dataset; these features are not intended to be used for the main task of the model [49].
  - Model inversion (attribute inference): The adversary aims to infer sensitive features used as input to the model [18].
2. Poisoning attacks: These attacks target the model and data integrity. The adversary maliciously alters the model through manipulating the raw data, or the model updates on the user side or as an external eavesdropper. These attacks aim to achieve one of the following goals.
  - Model corruption (label-flipping): The adversary corrupts the model to reduce its overall accuracy in its main task. This attack can target specific classes or be untargeted [50].
  - Backdoor: The adversary implants a backdoor sub-task in the model while maintaining good accuracy of the main task. This backdoor is used later in the production phase to exploit the model, e.g., by forcing misclassification of a specific input [16].

Our mapping shows in Figure 6 that the majority of the papers focus on privacy attacks—29 (60%)—and 19 (39%) focus on poisoning attacks. This may be explained by the fact that FL is mainly promoted as means of mitigation for several privacy risks [9]. Therefore, many researchers investigated the potentials and limitations of privacy in FL by crafting various attacks. Among the different types of attacks, the ones that dominate the research publications are the model inversion 18 (38%) and backdoor 13 (27%). Model inversion is one of the most severe attacks, since the adversary, in some cases, can fully reconstruct the client data. Backdoors are quite powerful in manipulating the model performance in the production phase, which might leave a long-term impact on the systems.

**Mode.** An adversary might act in two different modes.

1. Passive: The adversary attempts to learn from the observed information, without interrupting or deviating from the regular training process. This mode is widely common in privacy attacks [19,21].
2. Active: The adversary acts maliciously in the training process, e.g., they manipulate the training data or model updates. This mode is needed for poisoning attacks [16,17].

Figure 6 shows that 30 (62%) attacks are launched in the active mode, and 26 (54%) in the passive mode. Out of these attacks, 8 (17%) are applied in both modes. This distribution can be correlated with the capabilities of the adversary in the two modes; i.e., in the active mode, the adversary is more powerful—thus, a wider variety of attacks can be made.

**Observation.** The adversary’s ability to observe the parameters of the target model might vary among different attacks. We consider two possibilities.

1. Black-box: The adversary can query the model and thus knows the inference result of a particular input. However, it does not observe the model’s parameters [51].
2. White-box: The adversary can observe the model’s parameters [25]. This capability typically enables adversaries to carry out more sophisticated attacks.

As the model’s parameters are typically shared between the server and all the clients in FL, most of the attacks 44 (92%) assumed the white-box scenario. The black-box was considered only in 9 (19%) attacks.

**Access point.** The adversary might exist at different locations with different roles in the system. In FL, the adversary can be (1) a curious server, (2) a compromised client, or (3) an external eavesdropper (see Section 2.2).

Figure 6 illustrates that 44 (92%) attacks were conducted by clients, and only 15 (31%) attacks assumed the server to be malicious or curious; and 10 (21%) papers included attacks that can be carried out by external eavesdroppers. This reflects a keen interest in the attacks from the client side because these attacks are mainly facilitated by the distributed nature of the FL.

### 4.3. Common Evaluation Setups

The effectiveness of the proposed attacks is mostly demonstrated through an empirical evaluation. This evaluation needs to be extensive and comprehensive to provide sufficient evidence for the attack validity under specific settings. In this section, we examine the experimental settings commonly used for evaluating FL attacks by looking into four aspects, namely, target models, datasets, countermeasures, and implementation technologies.

**Target models.** We refer here to the joined ML model that is trained through the FL process, and thus targeted by the attack. The type of the model can vary, as FL by definition is not restricted to specific types. The attacks might be designed to target one or multiple model types, or they can be completely model-agnostic. On a high level, we can classify the target models in the literature into NN models and non-NN models.

**Figure 7:**
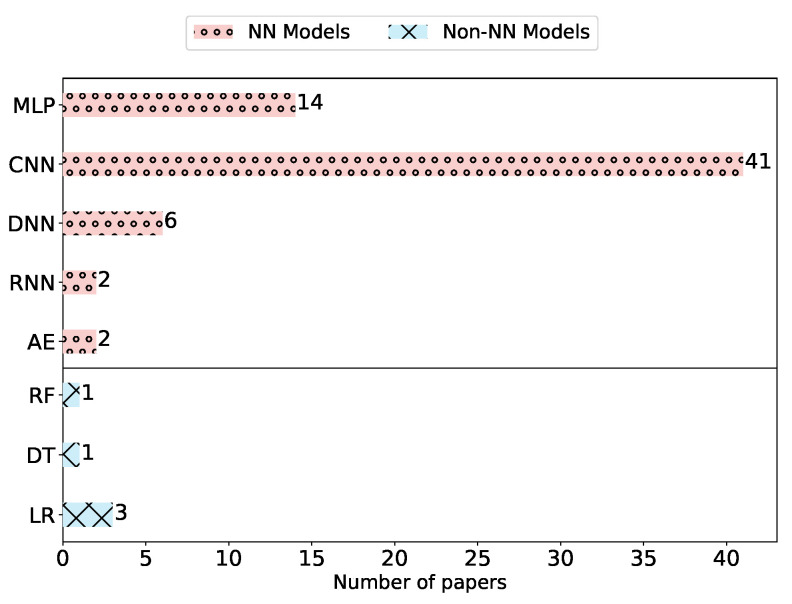
Target model types used to evaluate the attacks. Most of the papers evaluated their models against more than one other model.

The mapping results reveal that only three (6%) attacks targeted non-NN models. As shown in Figure 7, these three attacks consider logistic regression (LR) [51,52], and only one of them targets also decision tree (DT) and random forest (RF) [52]. Other attacks mainly focus on NN models, which can have diverse architectures (see Section 2.1). Interestingly, we can observe that the model type CNN was dominant as a target model in 41 (85%) attacks. This attention can be explained by the fact that CNNs are considered the state of the art for a wide variety of computer vision applications [53]. Other NN models such as Recurrent Neural Network (RNN) (e.g., Long Short-Term Memory) and Autoencoder (AE) (e.g., transformers) were the targets of attacks only twice in the literature. Furthermore, one attack was claimed to be model-agnostic [54].

**Datasets.** To train the target model, various datasets were used in the literature. These datasets can be categorized into three groups based on the type of the data: text, image, and key–value pairs. In total, 46 distinct datasets were used in the attack evaluations:

1. Text: CLiPS Stylometry Investigation, Yelp-author, Reddit, Amazon Review, Yahoo Answers.
2. Image: MNIST, Fashion-MNIST, LFW, CelebA, AT&T, CIFAR, CH-MNIS, ChestX-ray8, EndAD, EMNIST, Fer-2013, HAM10000, ImageNet, PIPA, SVHN, PubFig, Omniglot, mini-ImageNet, VGG2Face, fMRI, CASIA, Face, CINIC, Breast Histopathology Images.
3. Key–value: Purchase, BC Wisconsin, Adult, FourSquare, Human Activity Recognition, Landmine, Texas-100, UNSW-Benign, Parkinson Data, Yelp-health, Bank Marketing, Credit Card, Drive Diagnosis, News Popularity, KDDCup99, DIoT, Criteo.

**Figure 8:**
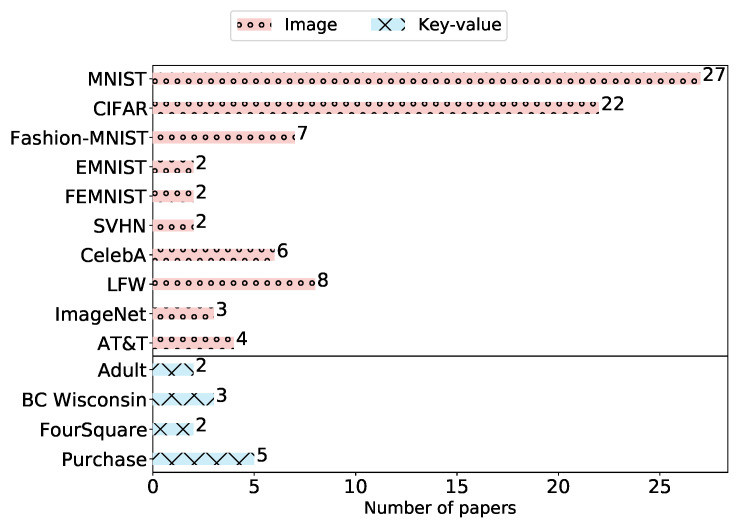
Datasets used more than once in the literature to evaluate the attacks. Most of the papers were evaluated using more than one dataset.

Over 72% of these datasets were used only once in the literature. The more prominent datasets are shown in Figure 8, where MNIST and CIFAR are the most common ones, used in 27 (56%) and 22 (46%) attacks, respectively. This conforms also with the common datasets in the ML community. The popularity of MNIST can be due to several reasons, e.g., its small size, such that researchers can train their models quickly and report results. In addition, it is widely supported, as is CIFAR, by many ML frameworks; thus, they can be easily used [55].

**Countermeasures.** One of the main methods to evaluate the proposed attacks is measuring their effectiveness against state-of-the-art defense mechanisms. We explored the mechanisms used in the examined papers; they can be classified into three main categories.

1. Perturbation: This mechanism reduces the information leakage about the clients in FL by applying one of the following perturbation techniques.
  - Noisy updates: A client may add noise to their data [56] or the updates before sending them to the server [19]. The noise can also be added on the server side [46]. The amount of noise can be carefully specified to achieve differential privacy.
  - Restricted updates: Before sharing the updates with the server, a client can limit the number of updates [57] or compress the updates, e.g., by applying quantization [58].
  - Regularization: While training the model locally on the client’s device, the client can apply regularization techniques such as dropout and batch normalization [59].
2. Cryptographic approaches: Exposing the updates of an individual client can lead to severe information leakage about their training data [19]. Several techniques based on cryptography are proposed to mitigate this risk.
  - Homomorphic encryption: Users can encrypt their updates with homomorphic encryption before sharing them with the server. Due to the homomorphic property, the server can compute the aggregation of the encrypted updates from all users to obtain an updated and encrypted global model. This model then is shared with the users, who can decrypt it [60].
  - Secret sharing: Users can encrypt their updates with keys derived from shared secrets. That is, the server needs to aggregate the encrypted updates and thus the shared secrets from a sufficient number of users in order to be able to decrypt the aggregate [45].
  - Trusted execution environment (TEE): The aggregation process on the server can be moved into a TEE, such that the executed code can be attested and verified to not leak individual clients’ updates [24].
3. Sanitization: This mechanism is proposed to mitigate poisoning attacks. In this respect, two defense mechanisms have been developed in the literature
  - Robust aggregation: To limit the impact of malicious updates on the global model, aggregation methods such as trimming the mean and calculating the median [61] are proposed.
  - Anomaly detection: Here, the malicious updates are assumed to be anomalies. To identify the anomalous updates (outliers), various techniques can be used, such as clustering [62] or measuring similarity with a reference set of samples [63].

**Figure 9:**
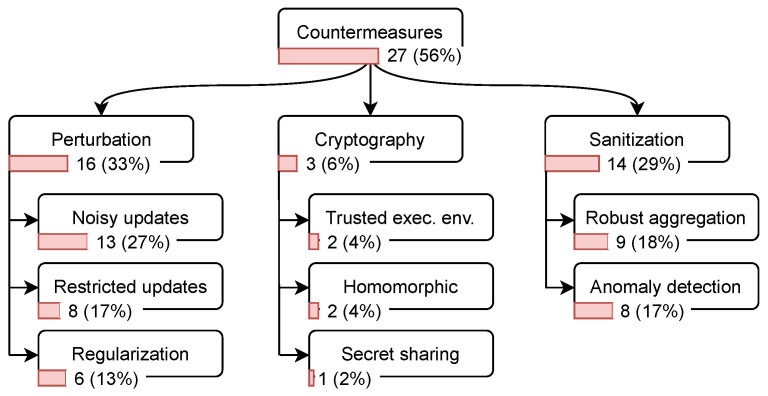
Countermeasures’ classification with the papers’ distribution. The percentage is with regard to the total number of papers, 48. Some categories are not exclusive; therefore, the papers might sum up to more than 48. More details on the individual papers can be found in Table 5. Only 55% of the attacks are evaluated against countermeasures. Noisy update is the most used technique with 27%.

The mapping results are depicted in Figure 9, where we can see that perturbation and sanitization are commonly used to evaluate attacks in FL: in 16 (33%) and 14 (29%) of the papers. This corresponds with the view that many researchers have about perturbation techniques (particularly, differential privacy) as the de facto standard for privacy-preserving ML [37]. Another reason for the high popularity of perturbation techniques could be that they were subject to intensive research not just in the FL community, but in the ML community in general. In contrast, sanitization is limited only to the FL setting. On the other hand, cryptography-based approaches are discussed in three (7%) papers. This could be because the security and privacy guarantees of these approaches were validated through formal proofs; thus, no empirical experiments are required.

It is worth mentioning that there is a large number of papers that propose constructive approaches for improving the security and privacy of FL under specific applications by using different technologies, e.g., blockchain [64,65]. We, however, do not cover this part of the literature here, since our focus is on the feasibility of existing attacks. We only focus on some of the proposed countermeasures that were used in the studied attacks’ papers to show against which countermeasures the assessed attacks have been evaluated by their authors.

**Implementation technologies.** To ease the reproducibility of the evaluation results, researchers are encouraged to share appropriate descriptions of their implementations, along with their source code [66]. In order to learn about the status of the selected papers in this respect, two factors were examined:

- Technology description: We checked whether the authors state clearly which technologies they use to implement their experiments, such as programming languages and libraries.
- Source code availability: We checked whether the source code has been made publicly available.

Table 4 shows the number of papers that reveal information about the technologies used in their implementation. A special notice can be put on the popularity of Python as a programming language and PyTorch as a specific Python package in this field, as shown in Table 4. The large share of Python-based implementations could be due to the fact that Python is easy to use and provides a large number of packages for ML tasks. PyTorch is user-friendly and suitable to creating custom models; for that and other reasons, it is widely used in the ML research community. On the other hand, 23 (48%) papers did not reveal any information about the technologies used in their implementations. Moreover, the mapping shows that the source code of only eight (16%) papers has been shared publicly.

**Table 3:** Mapping results for the studied papers with regard to meta data and attacks properties.

ID	Paper	Year	Venue	Affiliation	Type of Research	Attack Purpose	Attack Mode	Observation	Access Point
1	Hitaj et al. [18]	2017	C,R	A	S	MV	A	W	C
2	Bagdasaryan et al. [16]	2018	C,R	A	S	BD	A	W	C
3	Bhagoji et al. [17]	2018	W	A,I	S	BD	A	W	C
4	Bhagoji et al. [67]	2019	C,R	A,I	S	BD	A	W	C
5	Wang et al. [25]	2019	C,R	A	S	MV	A,P	W	S
6	Nasr et al. [27]	2019	C,R	A	S	MI	A,P	W,B	C,S
7	Zhu et al. [19]	2019	C,R	A	S	MV	P	W	C,S,E
8	Wang et al. [68]	2019	R	A	S	PI	P	W	C
9	Melis et al. [69]	2019	C,R	A	S	MI,PI	A,P	W	C
10	Mao et al. [70]	2019	C	A	S	MI,MV	A	W	C
11	Liu et al. [71]	2019	C,R	A	S	MI	P	W	C
12	Sun et al. [72]	2019	R	I	E	BD	A	W	C
13	Fang et al. [51]	2019	R	A	S	MC	A	W,B	C
14	Zhang et al. [73]	2019	C	A	S	BD	A	W	C
15	Mahloujifar et al. [54]	2019	C	A	S	BD	A	W	C,S
16	Tomsett et al. [74]	2019	C	I	S	BD	A	W	C
17	Cao et al. [75]	2019	C	A	E	BD	A	W	C
18	Baruch et al. [76]	2019	C,R	A	S	MC,BD	A	W	C
19	Fung et al. [77]	2019	R	A	S,E	MC,BD	A	B	C
20	Zhao et al. [20]	2020	R	A	S	MV	P	W	C,S,E
21	Wei et al. [78]	2020	R	A	E	MV	A	W	C,S,E
22	Pustozerova and Mayer [56]	2020	W	A	E	MI	P	W	C
23	Geiping et al. [79]	2020	R	A	S	MV	A,P	W	S,E
24	Sun et al. [80]	2020	R	A	S	MC	A	W	C
25	Nguyen et al. [81]	2020	W	A	S	BD	A	B	C
26	Chen et al. [63]	2020	C,R	A	S	BD	A	W	C
27	Song et al. [82]	2020	J	A	S	MV	A,P	W	S
28	Zhang et al. [21]	2020	C	A	S	MI	P	W	C
29	Zhang et al. [83]	2020	J	A	S	MC,BD	A	W	C
30	Tolpegin et al. [50]	2020	C,R	A	S	MC	A	W	C
31	Luo et al. [52]	2020	R	A	S	PI	P	W	C
32	Zhu et al. [84]	2020	R	A	S	PI	P	W	C,E
33	Mo et al. [85]	2020	R	A	S	MV	P	W	C
34	Wu et al. [86]	2020	C	A	S	MV	P	W	C,S,E
35	Wang et al. [87]	2020	R	A,I	S	MV	P	W	C,S,E
36	Xu et al. [88]	2020	C	A	E	PI	A,P	W	C
37	Chen et al. [89]	2020	C	A	E	MI	P	W	C
38	Lu et al. [90]	2020	R	A,I	E	MI	P	W	E
39	Xu et al. [91]	2020	C	A	E	MV	A	W	C
40	Qian et al. [92]	2020	R	A	E	MV	P	W	C,S,E
41	Xie et al. [93]	2020	C,R	A	E	MC	A,P	B	C
42	Wainakh et al. [94]	2021	C	A	S	MV	P	B,W	C,S,E
43	Shen et al. [95]	2021	J	A	E	MV	P	W	C,S
44	Enthoven et al. [22]	2021	R	A	S	MV	P	W	S
45	Fu et al. [96]	2021	R	A	S	MV	A,P	W	C
46	Shejwalkar et al. [23]	2021	R	A,I	S	MC	A	B,W	C
47	Wainakh et al. [97]	2021	R	A	S	MV	P	B,W	C,S,E
48	Shejwalkar et al. [98]	2021	R	A	S	MC	A	W	C

**Acronyms:** Venue: Conference (C), Public Repository (R), Journal (J), Workshop (W); affiliation: Academic (A), Industrial (I); Type of Research: Solution (S), Evaluation (E); attack purpose: Membership Inference (MI), Model Inversion (MV), Property Inference (PI), Model Corruption (MC), Backdoor (BD); attack mode: Active (A), Passive (P); observation: White Box (W), Black Box (B); Access Point: Server (S), User (U), Eavesdropper (E).

**Table 4:** Papers’ distribution with respect to reporting details on the implementation techniques.

Reported		Unreported
Python	23 (48%)	-
PyTorch	18 (38%)	
Public source code	8 (16%)	
Total	25 (52%)	23 (48%)

**Table 5:** Mapping results for the studied papers with regard to evaluation setups.

ID	Paper	Target Model	Num. of Datasets	Countermeasures	Public Code	Python	Libraries
1	Hitaj et al. [18]	CNN	2	NU	**✗**	**✗**	t7
3	Bagdasaryan et al. [16]	CNN,RNN	2	AD,NU,RA	**✗**	**✓**	pt
3	Bhagoji et al. [17]	CNN	1	**✗**	**✗**	?	**✗**
4	Bhagoji et al. [67]	CNN,MLP	2	RA	**✗**	?	**✗**
5	Wang et al. [25]	CNN	2	**✗**	**✗**	?	**✗**
6	Nasr et al. [27]	CNN,MLP	3	**✗**	**✗**	**✓**	pt
7	Zhu et al. [19]	CNN,AE	4	NU,RU,SS,HE	**✓**	**✓**	pt
8	Wang et al. [68]	CNN,MLP	4	RU,Reg	**✗**	**✓**	pt,sk
9	Melis et al. [69]	CNN	7	RU,Reg,NU	**✗**	?	**✗**
10	Mao et al. [70]	CNN,DNN	2	**✗**	**✗**	?	**✗**
11	Liu et al. [71]	CNN,AE	3	**✗**	**✗**	?	**✗**
12	Sun et al. [72]	CNN	1	NU,Reg	**✓**	**✓**	tf,tff
13	Fang et al. [51]	LR,CNN,DNN	4	AD	**✗**	?	**✗**
14	Zhang et al. [73]	CNN	2	**✗**	**✗**	**✓**	pt
15	Mahloujifar et al. [54]	**✗**	**✗**	**✗**	**✗**	?	**✗**
16	Tomsett et al. [74]	CNN	1	**✗**	**✗**	**✓**	pt
17	Cao et al. [75]	CNN	1	RA	**✗**	?	**✗**
18	Baruch et al. [76]	CNN,MLP	2	AD,RA	**✓**	**✓**	pt
19	Fung et al. [77]	CNN	4	AD,RA	**✗**	**✓**	sk
20	Zhao et al. [20]	CNN	3	**✗**	**✓**	**✓**	pt
21	Wei et al. [78]	CNN,MLP	5	NU,Reg	**✗**	?	**✗**
22	Pustozerova and Mayer [56]	MLP	1	NU	**✗**	?	**✗**
23	Geiping et al. [79]	CNN	3	**✗**	**✗**	?	**✗**
24	Sun et al. [80]	CNN	4	**✗**	**✗**	?	**✗**
25	Nguyen et al. [81]	RNN	3	NU,AD,Reg	**✗**	**✓**	pt
26	Chen et al. [63]	CNN	2	AD	**✗**	?	**✗**
27	Song et al. [82]	CNN	2	HE,RA,RU,TEE	**✗**	**✓**	kr
28	Zhang et al. [21]	**✗**	1	**✗**	**✗**	**✓**	pt,kr,tf,sk
29	Zhang et al. [83]	CNN	3	AD	**✗**	**✓**	pt
30	Tolpegin et al. [50]	CNN,DNN	2	AD	**✓**	**✓**	pt
31	Luo et al. [52]	LR,DT,RF,MLP	4	NU,Reg	**✗**	**✓**	pt,sk
32	Zhu et al. [84]	CNN,DNN	2	TEE	**✓**	**✓**	pt
33	Mo et al. [85]	CNN,DNN	3	**✗**	**✓**	**✓**	pt,th
34	Wu et al. [86]	CNN	3	NU,RU	**✗**	**✓**	tf
35	Wang et al. [87]	CNN,DNN	4	**✗**	**✗**	**✓**	pt
36	Xu et al. [88]	CNN	2	**✗**	**✗**	?	**✗**
37	Chen et al. [89]	CNN	2	**✗**	**✗**	**✓**	pt,kr,tf
38	Lu et al. [90]	MLP	2	**✗**	**✗**	?	**✗**
39	Xu et al. [91]	LR,MLP	2	**✗**	**✗**	**✓**	kr,tf,tff,f
40	Qian et al. [92]	CNN,MLP	6	NU	**✗**	?	**✗**
41	Xie et al. [93]	CNN	1	RA	**✗**	?	**✗**
42	Wainakh et al. [94]	CNN	2	**✗**	**✗**	**✓**	pt
43	Shen et al. [95]	CNN,MLP	4	**✗**	**✗**	?	**✗**
44	Enthoven et al. [22]	CNN,MLP	2	**✗**	**✗**	?	**✗**
45	Fu et al. [96]	CNN,MLP	6	NU,RU	**✗**	?	**✗**
46	Shejwalkar et al. [23]	CNN,MLP	3	RU,RA	**✗**	?	**✗**
47	Wainakh et al. [97]	CNN	4	NU,RU	**✗**	?	**✗**
48	Shejwalkar et al. [98]	CNN,MLP	4	RA	**✗**	?	**✗**

**Acronyms:** Target Model: Convolutional Neural Network (CNN), Multilayer Perceptron (MLP), Deconvolutional Neural Network (DNN), Recurrent Neural Network (RNN), Autoencoder (AE), logistic regression (LR), decision tree (DT), random forest (RF); Countermeasures: Noisy update (NU), Restricted Updates (RU), Regularization (Reg), Secret Sharing (SS), Homomorphic Encryption (HE), Robust Aggregation (RA), Anomaly Detection (AD), Matching Networks (MN); Libraries: PyTorch (pt), Torch7 (t7), Tensorflow (tf), TensorflowFederated (tff), Keras (kr), Scikit-learn (sk), Theano (th), Fate (f).

## 5. Discussion

In this section, first, we derive gaps in the research field from the mapping results. Second, we highlight several issues in the assumptions made in some papers. Third, we identify fallacies in the evaluation of the attacks and discuss their implications.

### 5.1. Main Research Gaps

**Figure 10:**
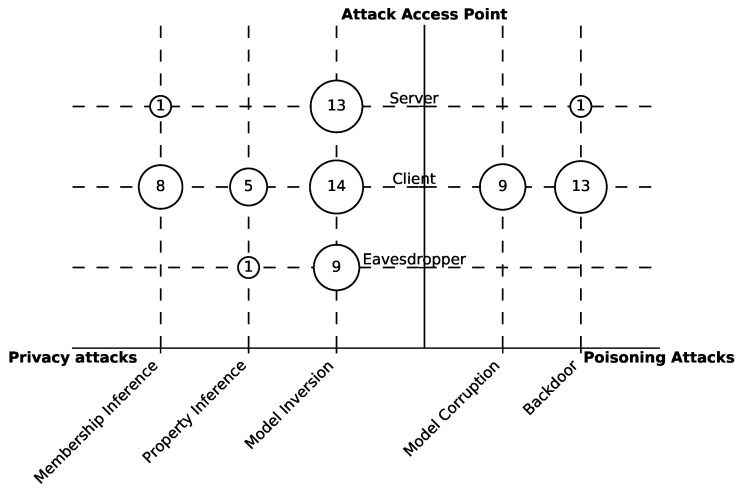
Bubble chart that shows the papers’ distribution on two dimensions: attack purpose and access point. Aside from model inversion, we notice a low number of attacks for the server and eavesdropper access point.

We base our discussion here on the results of Section 4. In addition, we are looking at how the papers are distributed over pairs of categories by the means of bubble charts, as shown for example in Figure 10, where we show how attacks with specific purposes are distributed with regard to the access point. It should be noted that the categories in some classification schemes are not disjoint; therefore, the total number of publications may sum up to more than 48.

**Gap 1.** Little research is conducted about attacks on the server side and by eavesdroppers.

*Description.*Figure 10 illustrates that membership, property inference, model corruption, and backdoor attacks are rarely studied on the server side or with an eavesdropper adversary. This might be due to two reasons. First, it is widely assumed in the literature that FL is coordinated by a trusted server. Second, approaches that protect against curious servers and eavesdroppers, such as secure aggregation [45], were proposed and widely adopted by the research community because of the firm protection guarantees they achieve. However, applying such approaches still incurs nonnegligible overhead [99], despite the improvements, which leaves open questions about their efficiency in real-world applications.

*Implications.* Typically, servers (service providers) are supposedly better equipped to repel attacks compared with clients. However, numerous events in recent years showed us that providers were subject to many successful attacks, where users’ data were breached. Therefore, it is of high importance to study how attacks by a curious or compromised server can impact the FL process. We argue that attacks on the server side are becoming even more relevant in FL, especially considering the emergence of applying FL in different architectures, such as hierarchies in edge networks [100,101]. In such environments, multiple entities play the role of intermediate servers, i.e., collect and aggregate the updates from clients, thereby introducing more server-type access points.

For eavesdroppers, recent model inversion attacks on gradients were proved to successfully reconstruct user training data [19,20]. This opens the door for more investigations about how gradients or model updates can be exploited to apply other attack types, especially privacy attacks.

**Gap 2.** Very little effort is devoted to studying attacks on ML functions other than classification.

*Description.* ML models can be used to fulfill a variety of functions, such as classification, regression, ranking, clustering, and generation. However, our SMS shows that there is a heavy bias towards the classification function: 46 (96%) of the attacks. Other functions, namely, regression, generation, and clustering, were addressed in only 4 (9%), 1 (2%), and 1 (2%) attacks, respectively.

*Implications.* This gap involves a lack of knowledge with respect to a large spectrum of models and applications that have different functions than classification. These functions are of high importance in many domains, e.g., ranking in natural language processing [102] and recommender systems [103]. It is an open question of how the existing attacks impact these functions. It is worth mentioning that a similar gap was also observed for adversarial attacks in general ML settings by Papernot et al. [28].

**Gap 3.** There is lack of research about attacks on ML models other than CNNs.

**Figure 11:**
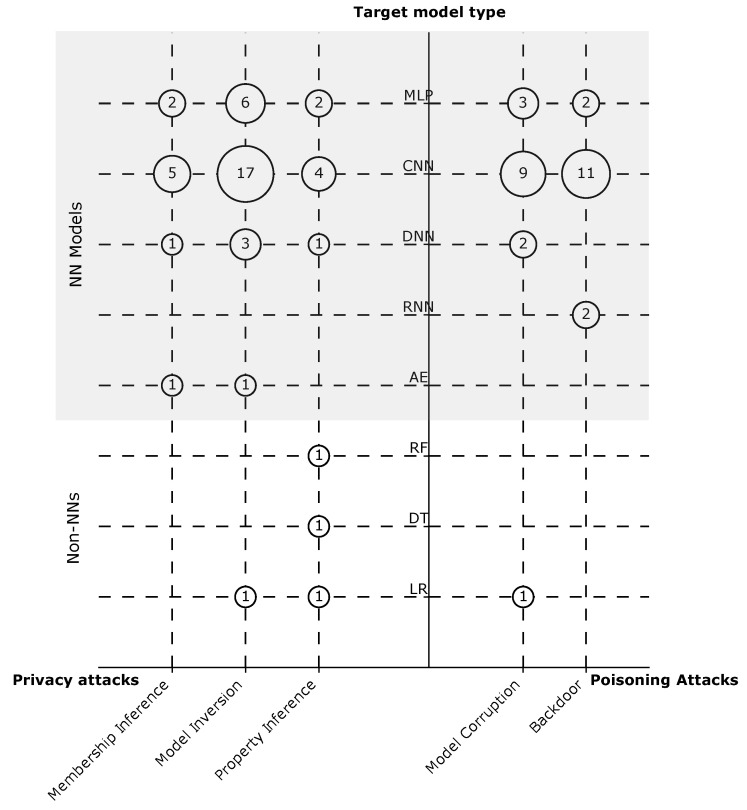
Bubble chart that shows the papers’ distribution in two dimensions: attack purpose and target model. We see a very low frequency of all attack types on non-NN models. Additionally, NN models such as Recurrent Neural Network (RNN) and Autoencoder (AE) receive little attention from various attacks.

*Description.* Although FL is not restricted to NN models, we have seen in the previous section that only three (6%) attacks target non-NN models. On a closer look, we depict in Figure 11 the types of models targeted by the different attacks. We notice that non-NN models were never targeted by membership inference or backdoor attacks. For NN models, we observe that RNN were not studied under any type of privacy attacks or model corruption attacks. Additionally, no research has been carried out on backdoors for DNNs. The AEs also have received very little attention. We only found only two privacy attacks using AEs. Overall, this illustrates the limited diversity in the literature considering the target models.

*Implications.* NN models are the state of the art in several applications, e.g., face recognition [104]; however, other ML models are still of high value and usage in real-world systems, e.g., genome analysis [105], culvert inspection [106], and filtering autocompletion suggestions [107], to name a few.

Within the NN models, there are a variety of network architectures, and as we show above, many of these architectures are not well covered in the evaluation of the attacks, even architectures that are widely used in several applications, e.g., RNN, which is used in Gboard [108]. Consequently, the evaluations of the proposed attacks fall short of providing evidence on how the attacks will perform against other network architectures.

Overall, we noticed limited effort devoted to studying the influence of using different model architectures on the effectiveness of the proposed attacks. We found only Geiping et al. [79] providing an adequate analysis of this aspect. Consideration of this issue when evaluating the attacks is important to improve the generalizability of the results.

### 5.2. Assumption Issues

There are a number of attacks that succeed only under special assumptions. These assumptions do not apply in many real-world scenarios; consequently, the applicability of these attacks is limited. Here, we highlight the issues of these assumptions and discuss their implications.

**Assumption Issue 1.** The attacks are effective only under special values of the hyper-parameters of the NN models.

*Description.* As described in Section 2, the hyper-parameters of NN models include, among others, the batch size, learning rate, activation function, and loss function. The hyper-parameters need to be carefully and fairly optimized to meet the application requirements. On contrary, we found in some papers that the hyper-parameters are tailored to demonstrate the high effectiveness of the attacks rather than to illustrate realistic scenarios.

*Examples and Implications.* In some model inversion attacks, the gradients are used to reconstruct the training data. Zhu et al. [19] and Wei et al. [78] showed that their attacks perform well only when the gradients are generated from a batch size <8. Zhao et al. [20] proposed an attack to extract the labels of the clients from gradients. However, the attack works only when the batch size is one, which is an exceptional and uncommon value. Hitaj et al. [18] also used a batch size of one to evaluate their attack on the At&T dataset.

Using small batches leads to a lack of accurate estimation of the gradient errors, which in turn causes less stable learning. Additionally, this requires more computational power to perform a large number of iterations, where gradients need to be calculated and applied every time to update the weights. While FL pushes the training to the client device, it is essential to consider the limited resources of the client devices. Therefore, the efficiency of the local training process is an important requirement. That is, having batches of very small values <8 increases the computational overhead and is therefore not preferable for FL applications.

Although it is insightful to point out the vulnerabilities that some special hyper-parameters might introduce, it is of high importance to discuss the relevance of these hyper-parameters to real-world problems.

**Assumption Issue 2.** The attacks succeed only when a considerable fraction of clients are malicious and participate frequently in the training rounds.

*Description.* In cross-device FL, a massive number of clients (up to $10^{10}$) form the population of the application. Out of these clients, the server selects a subset of clients (∼100 [107]) randomly for every training round to train the model locally and share their updates [9]. This random sampling is assumed to be uniform (i.e., the probability for a client to participate is 1/clientpopulation) to achieve certain privacy guarantees for clients, in particular, differential privacy [109]. Under these conditions, it is rather unlikely for a specific client to participate in a large number of training rounds (≫$\frac{\mathrm{total}\;\mathrm{number}\;\mathrm{of}\;\mathrm{rounds}}{\mathrm{client}\;\mathrm{population}}$) or consecutive ones. However, this was found as an assumption in a number of papers to enable some privacy and poisoning attacks. Furthermore, several attacks require a large number of clients to collude and synchronize in order to launch an attack, which also can be tricky to achieve in some cases.

*Examples and Implications.* Hitaj et al. [18] assumed that the adversary participates in more than 50 consecutive training rounds in order to carry out a reconstruction attack successfully. A stronger assumption was made by [83], namely, to have the adversary participating in all the rounds to poison the model. This requires the adversary to fulfill the FL training requirements [107] and to trick the server to be selected frequently, which is a challenge per se considering the setting described above.

State-of-the-art poisoning attacks in cross-device FL [51,76] assume up to 25% of the users to be malicious [23]. Considering that cross-device FL is mainly intended to be used by a massive number of users, the effective execution of these attacks would require the compromise of a significant number of devices. This in turn requires a very great effort and considerable resources, which could make the attacks impractical at scale [23]. For instance, a real-world FL application such as Gboard [108] has more than 1 billion users [110]. This means that the adversary ould need to compromise 250 million user devices to apply these attacks successfully [23]. However, it is worth mentioning that there are many ML applications (i.e., potential FL applications) on the market that use a smaller user base. Still, to the best of our knowledge, there are no real-world FL applications that represent this case.

The distributed nature of FL might indeed enable malicious clients to be part of the system. However, the capabilities of these malicious clients to launch successful attacks need to be carefully discussed in the light of applied FL use cases. Thus, the risk of these attacks is not overestimated.

**Assumption Issue 3.** The attacks can be performed when the data are distributed among clients in a specific way.

*Description.* FL enables clients to keep their data locally on their devices, i.e., the data remain distributed. This usually introduces two data properties: first, the data are non-IID; i.e., the data of an individual client are not representative of the population distribution. Second, the data are unbalanced, as different clients have different amounts of data [9]. In an ML classification task, for example, this may cause some classes not to be equally represented in the dataset. In any FL setting, it is essential to consider these two properties. While the meaning of IID and balanced data is clear, non-IID and unbalanced data distribution can be achieved in many ways [24]. In a number of papers, we found that specific distributions are assumed to enable the proposed attacks.

*Examples and Implications.* A backdoor attack on a classification model by Bagdasaryan et al. [16] achieved 100% accuracy on the backdoor task by one malicious client participating in one training round. However, in their experiment on CIFAR10, it was assumed that only the adversary has the backdoor feature, which is a big assumption [72]. The massive number of clients in FL suggests that the client’s data might cover the backdoor feature. Therefore, it should be considered that at least one honest client will have additional benign data for the backdoor feature.

Another example was found in the model inversion attack of [18], where the authors assumed that all data of one class belong to one client and that the adversary is aware of that. Additionally, their attack works only when all the data of one class are similar (e.g., images of one digit in the MNIST dataset). These assumptions do not apply to many real-world scenarios, so they were found to be unrealistic by [27]. Moreover, the model corruption attack introduced in [50] was launched under the setting of IID data, which contradicts the main FL assumptions. Similarly, Nasr et al. [27] evaluated their membership inference attack on a target model trained with balanced data. It is worth mentioning that Jayaraman et al. [111] pointed out the issue that most membership inference attacks [48,112,113] for stand-alone learning also focus only on the balanced distribution scenarios.

Overall, the way of implementing non-IID and unbalanced data distribution needs to be (1) discussed and justified in light of the application to assure as realistic as possible setup, (2) reflected clearly in the conclusions of the evaluation.

### 5.3. Fallacies in Evaluation Setups

Designing a comprehensive and realistic experimental setup is essential to prove the applicability of the attack and the generalizability of the conclusions. Although all the studied papers provide insightful evaluations of their proposed attacks, a number of practices were followed that might introduce fallacies. In this section, we set out to highlight this issue by identifying six fallacies. We discuss the implications of each fallacy on the evaluation results. Then, we propose a set of actionable recommendations to help to avoid them.

**Fallacy 1.** The datasets are oversimplified in terms of data content or data dimensions.

*Description.* Datasets are used to train and test the FL model and also to evaluate the attack. These datasets need to be representative of the population targeted by the model. As we highlighted in Section 4, the majority of attacks are evaluated on the image classification task. Therefore, here we focus on the image-based datasets.

Despite the growing calls for decreasing the usage of simple datasets, in particular MNIST [55], it is still one of the most common datasets in the deep learning community [114]. This is due to several reasons, such as its small size and the fact that it can be easily used in deep learning frameworks (e.g., Tensorflow, PyTorch) by means of helper functions [55].

MNIST was introduced by LeCun et al. [115] in 1998 and contains 70,000 gray-scale images of handwritten digits in the size of 28 × 28 pixels. Since then, substantial advances were made in deep learning algorithms and the available computational power. Consequently, MNIST became an inappropriate challenge for our modern toolset [116]. In addition, the complexity of images increased in modern computer vision tasks. That renders MNIST unrepresentative of these tasks [67].

Still, the phenomenon of the wide usage of MNIST is also observed in the examined papers: more than 53% (see Figure 8) used MNIST as the main dataset for evaluating the effectiveness of the proposed attacks. The second most common dataset was CIFAR, which is more complex in terms of data content; however, it is a thumbnail dataset; i.e., the images have are 32×32 pixels.

It is worth mentioning that in 41 (85%) of the papers, the authors evaluated their attacks on more than one dataset, which is a good practice. However, in a considerable number of papers (15 i.e., 31%), the authors used only datasets that contain either simple or small (thumbnail) images.

*Examples and Implications.* Using oversimplified datasets can lead to a misestimation of the attack capabilities. For instance, the capabilities of privacy attacks to retrieve information about the dataset are tightly related to the nature of this dataset. Consequently, the complexity and size of the images in the dataset impact the attacks’ success rate. It is clear that obtaining complex and bigger images requires higher capabilities. This is evident in the literature through several examples. Melis et al. [69] introduced a privacy attack that exploits the updates sent by the clients to infer the membership and properties of data samples. In [19], the authors demonstrated that the proposed attack of [69] only succeeds on simple images with clean backgrounds from the MNIST dataset. However, the attack’s accuracy degrades notably on the LFW dataset and fails on CIFAR. In the same context of privacy attacks, Zhu et al. [19] proposed the model inversion attack DLG, which reconstructs the training data and labels from gradients. Their experiments showed that DLG can quickly (within just 50 iterations) reconstruct images from MNIST. However, it requires more computational power (around 500 iterations) to succeed against more complex datasets such as CIFAR and LFW. Recently, Wainakh et al. [94] demonstrated that the accuracy of DLG in retrieving the labels degrades remarkably on CelebA, which has a bigger image size than the thumbnail datasets, such as MNIST and CIFAR.

*Recommendations.* We acknowledge that it is challenging to find a single dataset that provides an adequate evaluation of the attacks; therefore, it is essential to evaluate the attack on diverse datasets with regard to image complexity and dimensions. We encourage researchers to also consider real-life datasets, which pose realistic challenges for the models and attacks, e.g., ImageNet (image classification and localization) [117], Fer2013 (facial recognition) [118], and HAM10000 (diagnosing skin cancers) [119].

**Fallacy 2.** The datasets are not user-partitioned, i.e., not distributed by nature.

*Description.* In FL, data are distributed among the clients; each client typically generates their data by using their own device. Therefore, these data have individual characteristics [9]. The datasets used for evaluating the attacks should exhibit this property, i.e., be generated in a distributed fashion. However, in only 11 (23%) of the papers were user-partitioned datasets used. One of these datasets is EMNIST [120], which was collected from 3383 users, and thus, it is appropriate for the FL setting [72]. Researchers in the majority of studies, (37 i.e., 77%), used pre-existing datasets that are designed for centralized machine learning [121] and thus are unrealistic for FL [122]. These datasets then were artificially partitioned to simulate the distributed data in FL. One additional issue with these datasets is that they are by default balanced, yet FL assumes the client’s data to be unbalanced [9].

*Examples and Implications.* In the image classification use case, the poisoning attacks proposed in [16,67] were evaluated on centralized datasets, such as Fashion-MNIST and CIFAR. The attacks were reported to achieve 100% accuracy in the backdoor task. However, by using EMNIST as a standard FL dataset, Sun et al. [72] illustrated the limitations of the previous attacks. More precisely, they showed that the performance of the attacks mainly depends on the ratio of adversaries in the population. Moreover, the attacks can be easily mitigated with norm clipping and “weak” differential privacy. Although this fallacy was discussed in previous works [121,122], its implications on the evaluation results need to be investigated further and demonstrated with empirical evidence.

*Recommendations.* It is recommended to use FL-specific datasets for adequate evaluation of the attacks. Researchers have recently been devoting more efforts to curating such datasets. The LEAF framework [122] provides five user-partitioned datasets of images and text, namely, FEMNIST, Sent140, Shakespeare, CelebA, and Reddit. Furthermore, Luo et al. [121] created a street dataset of high-quality images, which is also distributed by nature for FL.

**Fallacy 3.** The attacks are evaluated against simple NN models.

*Description.* We observe a major focus on attacking NN models in federated settings. These models can have a variety of architectures, as discussed in Section 2. The complexity of these architectures vary with respect to the number of layers (depth), the number of neurons in each layer (width), and the type of connections between neurons. In the case of CNN models (41 papers), our study shows that researchers tend to use simple architectures to evaluate their attacks (21 (52%) papers), e.g., 1-layer CNN [22] and 3-layer CNN [67]. In 20 (49%) papers, the authors considered complex state-of-the-art CNN models, such as VGG [123], ResNet [124], and DenseNet [125], the winners of the famous *ImageNet Large Scale Visual Recognition Challenge (ILSVRC)* [126].

*Examples and Implications.* It is reasonable to start evaluating novel attacks on simple models to facilitate the analysis of the initial results. However, this is insufficient for drawing conclusions on the risks posed by these attacks to real-life FL-based applications for two reasons. First, modern computer vision applications, e.g., biometrics, use advanced models, mostly with sophisticated architectures, to solve increasingly complex learning objectives [127]. Second, in deployed systems, a ML model typically interacts with other components, including other models. This interaction can be of extreme complexity, which might introduce additional challenges for adversaries [128]. For instance, in the Gboard app [108], as a user starts typing a search query, a baseline model determines possible search suggestions. Yang et al. [107] utilized FL to train an additional model that filters these suggestions in a subsequent step to improve their quality.

Several model inversion attacks reconstruct the training data by exploiting the shared gradients [22,78,97]. In particular, they exploit the mathematical properties of gradients in specific model architectures to infer information about the input data. For example, Enthoven et al. [22] illustrated that neurons in fully connected layers can reconstruct the activation of the previous layer. This observation was employed to disclose the input data in fully connected models with high accuracy. However, the same attack achieves considerably less success when the model contains some convolutional layers.

The NN capacity (i.e., number of neurons) also influences the performances of some attacks, in particular, backdoors. It is conjectured that backdoors exploit the spare capacity in NNs to inject a sub-task [129]. Thus, larger networks might be more prone to these attacks. However, this interesting factor still needs to be well investigated [72]. In this regard, it is worth mentioning that increasing the capacity, e.g., for CNNs, is a common practice to increase the model accuracy. However, recent approaches, such as EfficientNet [130], call for scaling up the networks more efficiently, achieving better accuracy with smaller networks. This development in the CNNs should be also considered in the evaluation of the attacks.

*Recommendations.* We highly encourage the researchers to consider the state-of-the-art model architectures that are widely used in the applications where they will apply their attacks. In addition, it would be insightful for a more realistic security assessments to consider evaluating the proposed attacks on deployed systems that contain multiple components.

**Fallacy 4.** The attacks are designed for cross-device scenarios (massive client population), yet evaluated on a small number of clients ≤100.

*Description.* FL can be applied in cross-silo or cross-device settings. In the cross-silo setting, clients are organizations or data centers (typically 2–100 clients), whereas in the cross-device scenario, clients are a very large number of mobile or IoT devices (massive up to $10^{10}$) [24]. For instance, in applied use cases of FL, Hard et al. [108] reported using 1.5 million clients to train the Coupled Input and Forget Gate language model [131]. Yang et al. [107] trained a logistic regression model (for the Gboard application) for 4000 training rounds. They employed 100 clients in each round.

Although many of the studied papers do not explicitly use the term “cross-device” to describe their scenario, they refer mainly to clients as individual users who have personal data. However, 27 (56%) papers provided an evaluation with a total population of ≤100 clients. Moreover, 13 (27%) of the papers did not report at all the client population in their experiments.

*Examples and Implications.* The total number of clients and the clients participating per round in FL determine the influence of a single client on the global model. For privacy attacks, this means that each client contributes considerably to shaping the model parameters; thus, the parameters more prominently reflect the client’s personal data. Shen et al. [95] demonstrated that increasing the client population led to a decrease in the accuracy of their property inference attack. For poisoning attacks, using a small number of clients amplifies the impact of the poison injected by malicious ones. This was shown in the experiments of [67], where the accuracy of the backdoor task degraded with bigger client populations.

*Recommendations.* We recommend researchers to consider a large number of clients to evaluate novel attacks. For that, it is helpful to use the datasets provided by LEAF [122], which contain more than 1000 clients. In case large-scale evaluation is not feasible, researchers are encouraged to discuss at least the potential implications of different client populations on their attacks.

**Fallacy 5.** The attacks are not evaluated against existing defense mechanisms.

*Description.* An attack becomes ineffective if it requires the adversary to make a disproportional large effort to overcome a small defense mechanism [128]. Proposed attacks need to be evaluated in this respect with state-of-the-art defenses. However, we showed in Section 4.3, Figure 9, that 21 (48%) of the proposed attacks were not evaluated against any of the defense mechanisms. In most of these papers, the authors only discussed theoretically potential countermeasures to mitigate their attacks.

**Figure 12:**
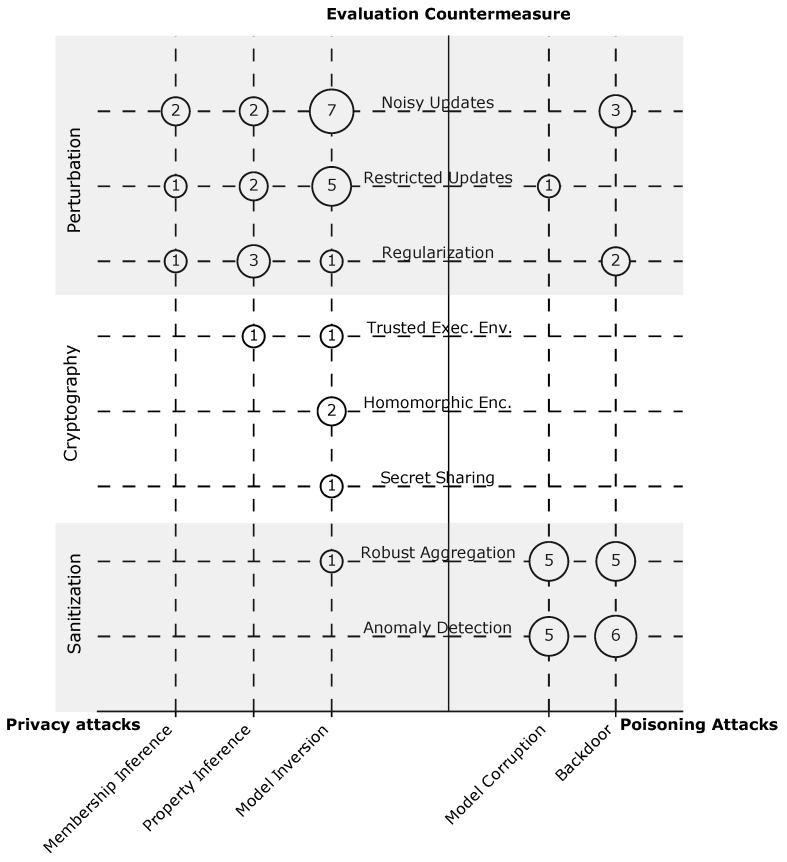
Bubble chart that shows the papers’ distribution in two dimensions: attack purpose and countermeasures. We see that perturbation and cryptography-based countermeasures are mainly used for privacy attacks, and sanitization is used for poisoning attacks.

*Examples and Implications.* This fallacy leaves the evaluation of the attacks incomplete, and their applicability under real-world scenarios, where defense mechanisms are typically deployed, is questionable. However, it is important here to distinguish between the different categories of defense mechanisms. On the one hand, cryptography-based defenses typically provide formally proved properties; thus, in some cases, their impacts on the attacks can be sufficiently discussed without empirical evidence. Still, in these cases, efficiency remains an open question. On the other hand, the impacts of other defense categories, namely, perturbation and sanitization, against attacks require experimental analysis, as these defenses usually introduce loss in the model accuracy, hence need to be customized to reach a desired balance between the accuracy and privacy. In Figure 12, we see that most of the implemented defenses in the literature are from these two categories. We see also that perturbation is mainly used for privacy attacks, which reduces the information leakage about individuals, whereas sanitization mitigates the impact of malicious updates from adversaries, and thus is used against poisoning attacks.

*Recommendations.* We highly recommend evaluating novel attacks against the appropriate state-of-the-art defenses. For implementing perturbation approaches, emerging libraries such as Opacus (https://github.com/pytorch/opacus) and Tensorflow Privacy (https://github.com/tensorflow/privacy) can be used.

**Fallacy 6.** The results of the experimental evaluations are not easily reproducible.

*Description.* The majority (97%) of the proposed attacks are validated through empirical experiments. To accurately reproduce the results of these experiments by other researchers, several practices need to be considered. In our analysis, we took into account three main practices: (1) using publicly available datasets, (2) reporting technical details about the implementation, and (3) publishing the source code. Our study shows in Section 4.3 that public datasets were used in all the examined papers, which is a good practice. However, 23 (48%) papers did not contain any details about the technologies used in the implementation. Furthermore, the source code of 40 (83%) papers was not found publicly.

*Examples and Implications.* Dacrema et al. [66] reported that reproducibility is one of the main factors to assure progress for research, especially with approaches based on deep learning algorithms. To conduct a proper assessment of a novel attack, researchers usually compare it with previous attacks as baselines. Evaluating the different attacks under different settings and assumptions hinders this direct comparison. That is, researchers have to re-implement the respective attacks to reproduce their results under different settings. This becomes even more challenging when the authors do not describe their experimental setups and parameters to the extent of full reproducibility.

*Recommendations.* We encourage all researchers to share their source code and detailed descriptions of their setups. We also recommend using libraries and benchmark frameworks that support FL, namely, Tensorflow-federated, PySyft [132], LEAF [122], FATE [133], and FedML [134]. This in turn will help researchers to implement their ideas more easily and improve the consistency of implementations and experimental settings across different papers.

## 6. Conclusions

In this paper, we carried out a systematic mapping study based on recent publications that address attacks in the federated learning setting. For that, we analyzed 48 relevant papers. We structured these papers in classification schemes regarding attacks and evaluation settings.

The results of our analysis showed that most of the work focuses on classification as a ML function, and little attention is paid to other functions, e.g., generation and clustering. Neural Network models, especially Convolutional Neural Networks, are intensively studied in the literature, whereas other ML algorithms and model types are not well covered. We additionally examined the assumptions of the proposed attacks to identify those with restricted applicability in the context of real-world scenarios. These assumptions range from choosing unorthodox values of hyper-parameters to constructing special kinds of data distribution among clients. We further identified six fallacies in the evaluation of the attacks which affect the generalizability of the results and led to overestimating the effectiveness of the attacks. For instance, the usage of overly simple or centralized datasets was found in the majority of the publications. Moreover, close to half of the attacks were proposed without considering the state-of-the-art defense mechanisms. Notably, there is ambiguity regarding reproducible research. As a constructive step, we presented several actionable recommendations to mitigate these identified fallacies by using modern models—federated-learning-specific datasets and frameworks.

Overall, our study revealed that each of the examined papers contains at least one of the assumption issues or is affected by one of the evaluation fallacies. Thus, the effectiveness of the attacks in real-world scenarios needs to be further investigated and supported by empirical evidence. However, we do not downplay the vulnerabilities and threats in federated learning discussed in the literature. Instead, our findings contribute to a more informed assessment of the severity of these vulnerabilities. This is key, with federated learning being a very promising ML setting in the context of smart sensor and IoT environments in order to mitigate users’ privacy concerns. We hope that our analysis will raise awareness of the common issues in the literature, and help researchers in orienting their future research by better understanding the current research progress in the domain of the security and privacy of federated learning.

## Figures and Tables

**Table 2:** Manual search sources.

Journal	Name	Publisher *
TOPS	Transactions on Privacy and Security	ACM
TIFS	Transactions on Information Forensics and Security	IEEE
**Conference**	**Name**	Publisher *
S&P	Symposium on Security and Privacy	IEEE
CCS	Conference on Computer and Communications Security	ACM
USENIX Security	USENIX Security Symposium	USENIX
PETS	Privacy Enhancing Technologies Symposium	Sciendo
EuroS&P	European Symposium on Security and Privacy	IEEE
NDSS	Network and Distributed System Security Symposium	Internet Society
CSF	Computer Security Foundations Symposium	IEEE
ACSAC	Annual Computer Security Applications Conference	ACM
ESORICS	European Symposium on Research in Computer Security	Springer
NeurIPS	Neural Information Processing Systems	Curran Associates
ICML	International Conference on Machine Learning	PMLR
ICLR	International Conference on Learning Representations	OpenReview.net
InfoCom	International Conference on Computer Communications	IEEE
AISTATS	Artificial Intelligence and Statistics	-

* The publishers list was checked for 2019.

## Data Availability

Data are available upon request to the authors.

## Abbreviations

The following abbreviations are used in this manuscript:

AE	Autoencoder
CIFG	Coupled Input and Forget Gate
CL	Collaborative learning
CNN	Convolutional Neural Network
DNN	Deconvolutional Neural Network
DOSN	Decentralized Online Social Network
DP	Differential Privacy
DT	decision tree
FL	Federated learning
HFL	Hierarchical federated learning
IID	Independent and Identically Distributed
IoT	Internet of Things
LR	logistic regression
LSTM	Long Short-Term Memory
ML	Machine learning
MLP	Multilayer Perceptron
NN	Neural Network
OSN	Online Social Network
RF	random forest
RNN	Recurrent Neural Network
SLR	systematic literature review
SMS	systematic mapping study
TEE	Trusted execution environment
